# Supplementary material for: Preclinical Efficacy of Peripheral Nerve Regeneration by Schwann Cell-like Cells Differentiated from Human Tonsil-Derived Mesenchymal Stem Cells in C22 Mice
Source: Biomedicines. 2023 Dec 17;11(12):3334. doi: 10.3390/biomedicines11123334 (PMC10741921; doi:10.3390/biomedicines11123334)
Supplement: Supplementary file 1 [file biomedicines-11-03334-s001.zip › biomedicines-2675364-supplementary.pdf]

Supplementary Figure S1

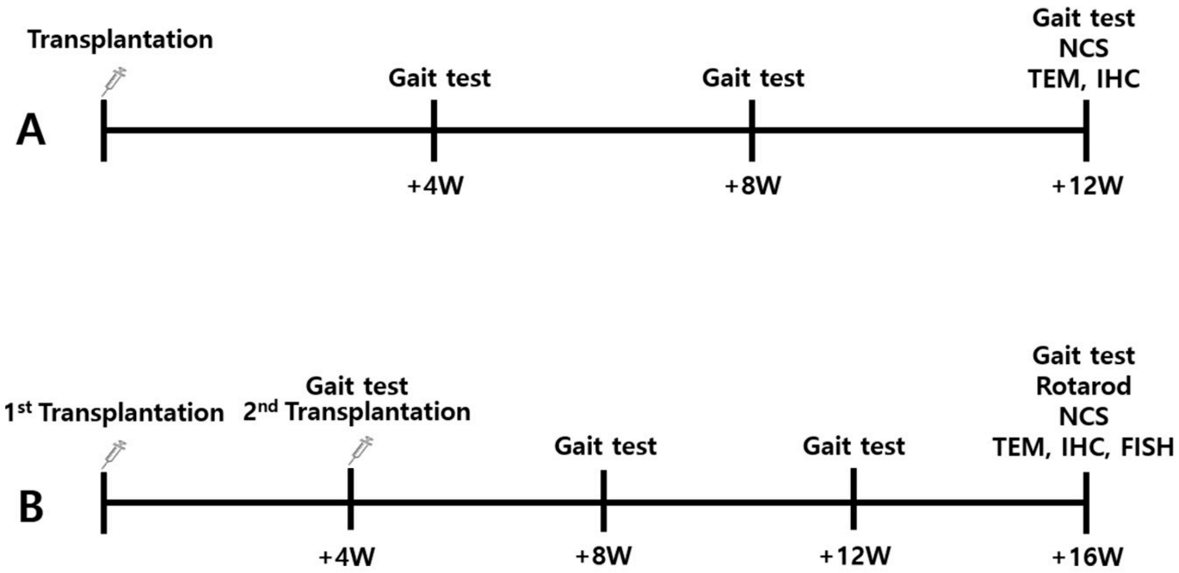

Figure S1. Schematic diagram of experiment for assessment of efficacy after transplantation with NRPCs in C22 mice. (A) Single-dose transplantation. (B) Repeated-dose transplantation. The repeated-dose was transplanted twice with a 4-week interval between each transplantation. The NRPC groups are the experimental group injected intramuscularly into both legs with 2.5×10^4 (low), 2.5×10^5 (medium), and 5×10^5 (high) cells in 100 µL CS10, respectively per thigh. NCS, nerve conduction study; TEM, transmission electron microscopy; IHC, immunohistochemistry; WB, western blot; FISH, fluorescence in situ hybridization; NRPC, neuronal regeneration-promoting cell.

Supplementary Figure S2

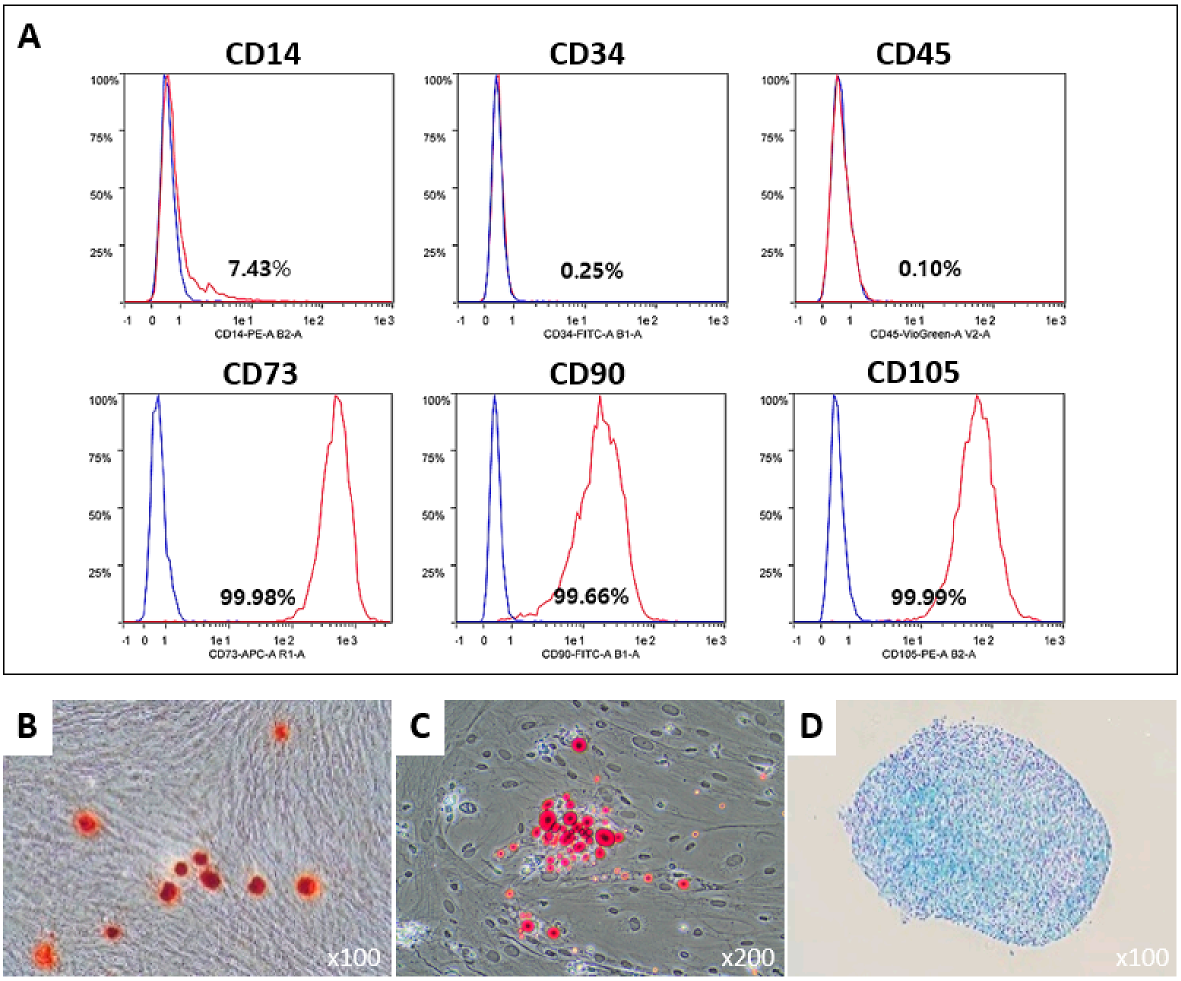

Figure S2. Characterization of human TMSCs as MSCs. (A) The profiles of MSC specific surface markers of TMSCs were analyzed by flow cytometry using CD14, CD34, CD45, CD90, CD73, and CD105. The blue histogram profiles indicate the isotype control, and the red histograms indicate a specific antibody. Establishment of the differentiation potential of TMSCs toward mesodermal lineages. TMSCs readily differentiated into osteoblasts, adipocytes, and chondrocytes, as stained by Alizarin Red (B), Oil Red (C), Safranin (D). MSCs, mesenchymal stem cells; TMSCs, tonsil-derived MSCs

Supplementary Figure S3

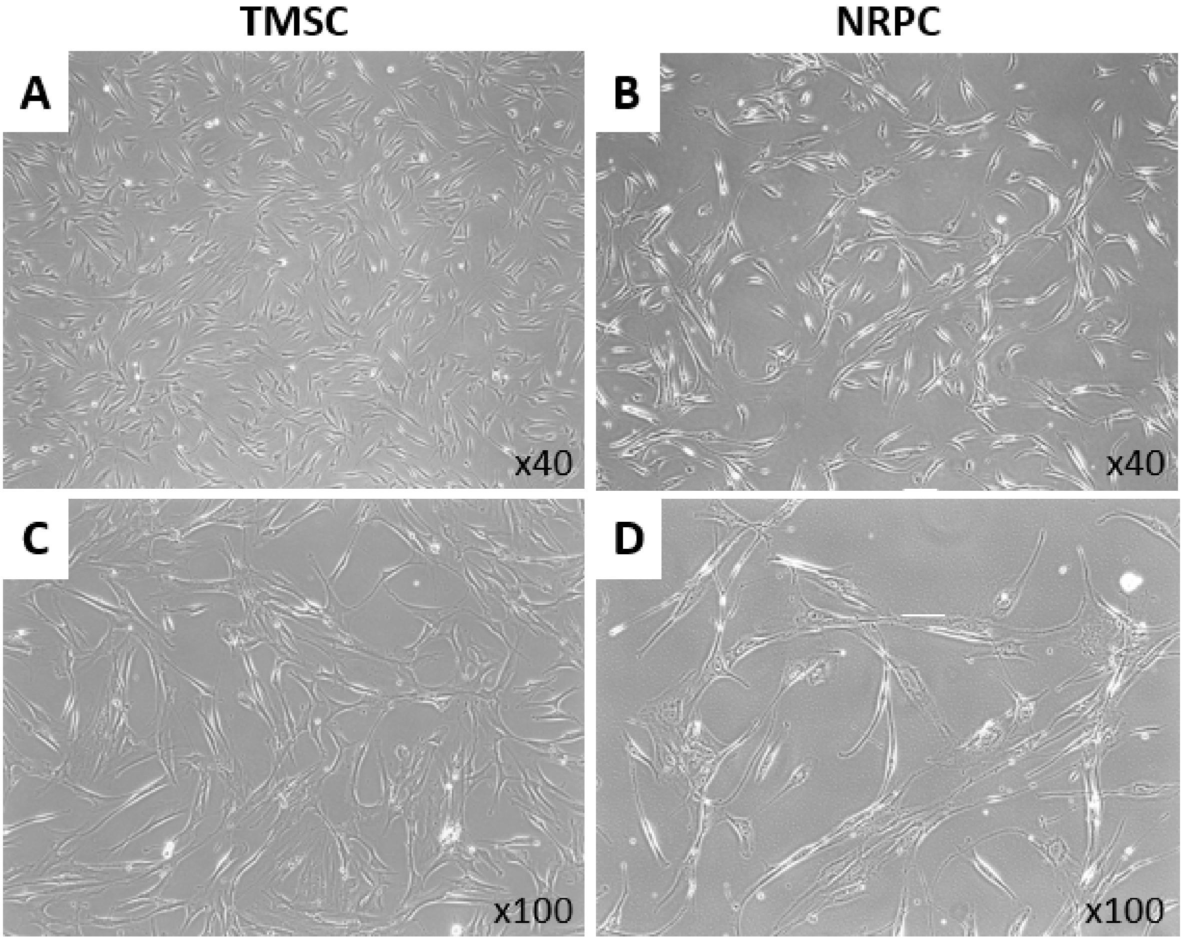

Figure S3. Differentiation of NRPCs derived from TMSCs. (A, C) TMSCs were observed under an inverted microscope (x40, x100). (B, D) NRPCs were observed under an inverted microscope (x40, x100). NRPCs exhibited morphological changes after differentiation, such as an elongated bipolar or tripolar spindle shape, with thinner cytoplasmic extensions and larger nuclei than TMSCs. TMSC, tonsil-derived MSC; NRPC, neuronal regeneration-promoting cell.

Supplementary Table S1

Table S1. Antibody information used for immunohistochemistry.

Name	Dilution	Vendor	Catalog No.
Rabbit anti-neurofilament heavy (NF-H)	1: 50	Santa Cruz	sc-20112
Chicken anti-myelin basic protein (MBP)	1: 50	Sigma-Aldrich	AB9348
Mouse anti-myosin heavy chain 8 (MYH8)	1: 50	DSHB	N3.36
Mouse anti-myosin heavy chain 1 E (MYH1E)	1: 50	DSHB	MF 20
Rabbit anti-laminin	1: 50	Novus Biologicals	NB300-144
Rabbit anti-peripheral myelin protein 22 (PMP22)	1: 50	Novus Biologicals	NBP2-67068
Rabbit anti-myelin protein zero (MPZ)	1: 50	Abcam	ab183868
Goat anti-rabbit IgG (H+L) cross-adsorbed secondary antibody, Alexa Fluor 568	1: 100	Invitrogen	A-11011
Goat anti-chicken IgY (H+L) secondary antibody, Alexa Fluor 488	1: 100	Invitrogen	A-11039
Goat anti-mouse IgG, IgM (H+L) secondary antibody, Alexa Fluor 488	1: 100	Invitrogen	A-10680
Goat anti-rabbit IgG (H+L) cross-adsorbed secondary antibody, Alexa Fluor 488	1: 100	Invitrogen	A-11008
